# The Response of Photosynthetic Functions of F_1_ Cutting Seedlings From *Physocarpus amurensis* Maxim (♀) × *Physocarpus opulifolius* “Diabolo” (♂) and the Parental Seedlings to Salt Stress

**DOI:** 10.3389/fpls.2018.00714

**Published:** 2018-06-04

**Authors:** Xu Nan, Zhang Huihui, Zhong Haixiu, Wu Yining, Li Jinbo, Xin Li, Yin Zepeng, Zhu Wenxu, Qu Yi, Sun Guangyu

**Affiliations:** ^1^Natural Resources and Ecology Institute, Heilongjiang Academy of Sciences, Harbin, China; ^2^College of Life Sciences, Northeast Forestry University, Harbin, China; ^3^College of Resources and Environment, Northeast Agricultural University, Harbin, China; ^4^College of Horticulture, Shenyang Agricultural University, Shenyang, China

**Keywords:** *Physocarpus amurensis* Maxim, *Physocarpus opulifolius* “Diabolo”, hybrid, salt stress, photosynthetic characteristics, chlorophyll fluorescence characteristics

## Abstract

This paper selected clonal cutting seedlings from the F_1_ hybrid varieties of *Physocarpus amurensis* Maxim (♀) × *P. opulifolius* “Diabolo” (♂) as research material to study the response of the photosynthetic gas exchange parameters and chlorophyll fluorescence parameters of *P. amurensis* hybrids and their parental leaves to NaCl stress (with concentrations of 0, 50, 100, and 200 mmol⋅L^-1^). The results showed that under salt stress, the stomatal conductance (*G*_s_), transpiration rate (*T*_r_), and net photosynthetic rate (*P*_n_) of the three kinds of *P. amurensis* all significantly decreased. When the NaCl concentration was below 100 mmol⋅L^-1^, the intercellular CO_2_ concentration (*C*_i_) of leaves of the three samples declined with the increase of salt concentration; however, when the concentration increased to 200 mmol⋅L^-1^, *C*_i_ did not decrease significantly, especially when the *C*_i_ of *P. opulifolius* “Diabolo” presented a slight increase. This indicated that the decline of photosynthetic carbon assimilation capacity induced by salt stress was the consequence of interaction between stomatal factors and non-stomatal factors, and the stomatal factors played an important role when the salt concentration was below 200 mmol⋅L^-1^. Compared with *P. amurensis*, the photosynthetic gas exchange capability of *P. opulifolius* “Diabolo” leaves was more sensitive to salt stress, and the limitation of non-stomatal factors was relatively evident. However, the photosynthetic capacity of hybrid *P. amurensis* leaves with the desired purple color was improved compared with *P. amurensis*. Under salt stress, the PSII activity of the three kinds of *P. amurensis* leaves declined, the electron transfer was inhibited, and obvious signs of photoinhibition were present. The PSII activity of *P. opulifolius* “Diabolo” leaves was more sensitive to salt stress than that in *P. amurensis*. Under salt stress, the NPQ of *P. opulifolius* “Diabolo” leaves decreased greatly, while under high salt concentrations the degree of photoinhibition in *P. amurensis* and hybrid *P. amurensis* were reduced due to a relatively high NPQ. With the increase of salt concentration, the *V*_k_ of *P. amurensis* and hybrid *P. amurensis* leaves presented a decreasing trend. However, the *V*_k_ of *P. opulifolius* “Diabolo” leaves increased slightly. This suggested that the effects of salt stress on the oxygen-evolving complex (OEC) of the three *P. amurensis* sample types were relatively limited and only the OEC of *P.s opulifolius* “Diabolo” leaves were slightly sensitive to salt stress. The *V*_J_ of all leaves from the three *P. amurensis* types increased under salt stress, and the *V*_J_ increased significantly when the salt concentration increased to 200 mmol⋅L^-1^, indicating that salt stress obviously impeded the electron transfer chain from *Q_A_* to *Q_B_* on the PSII receptor side. Moreover, high salt concentrations caused thylakoid membrane dissociation. The electron transfer and degree of damage to the thylakoid membrane of *P. opulifolius* “Diabolo” leaves were obviously higher than that of *P. amurensis*. However, the electron transfer capacity on the PSII receptor side as well as the degree of damage of the thylakoid membrane of hybrid *P. amurensis* leaves was obviously lower than those of *P. opulifolius* “Diabolo.” The salt tolerance of photosynthetic functions of hybrid *P. amurensis* (♀) × *P. opulifolius* “Diabolo” (♂) leaves was improved compared with that of parental *P. opulifolius* “Diabolo,” and the hybrid shows obvious hybrid vigor for photosynthesis.

## Introduction

Salt stress is one of the important factors limiting plant growth and development, especially under the current expansion of salt alkaline lands and gradual deterioration of the degree of salinity. Therefore, developing salt tolerant hybrids is vital to soil remediation with salt-alkaline vegetation as well as urban afforestation. Besides the direct toxic effect of salt ions to plants, salt stress will also result in osmotic stress and oxidative damage (Munns, 2002; Liang and Wang, 2009). Salt stress is one of the most severe environmental threats to plant growth and crop yield (Greenway and Munns, 1980). The standard salinity in soil had direct and indirect effects on plants. First, excessive soluble salts in soil solution can reduce water uptake by plants, restrict root growth, and cause osmotic stress. Second, high toxic ions (especially Na^+^) accumulated in plants can trigger ion poisoning and deficient nutrient elements (Zhu, 2001; Munns and Tester, 2008; Wang et al., 2008). These effects are closely related with the absorption of mineral salts by plants, the accumulation and distribution of mineral salts in plants. Therefore, the plants’ salt tolerance depended on their ability of absorbing, transporting, accumulating and distributing mineral salts. Under salt stress, the high K^+^/Na^+^ levels mean that the plants are more tolerant to salts. Shabala and Cuin (2008) pointed out that maintaining higher K^+^/Na^+^ levels in cytoplasm was much more important than simply maintaining low Na^+^ level and high K^+^ absorption. For this reason, whether plants can survive under salt infiltration largely depends on their ability to keep K^+^/Na^+^ balance under salt stress. The plant can maintain an effective K^+^/Na^+^ balance in cytoplasm by restricting Na^+^ absorption, increasing Na^+^ discharge, energizing Na^+^ segmentation in the vacuole and reducing K^+^ loss (Shabala and Cuin, 2008). Hybrid vigor is a ubiquitous biological phenomenon (Huang et al., 2016; Li et al., 2016). Numerous studies have shown that the growth of hybridal plants, photosynthetic capacity, and the adaptability to stress were all better than those of parental plants (Ye and Wang, 2002; Zhang et al., 2017) explained in the photosynthetic superiority hypothesis of the forest hybrid vigor that the origin of forest hybrid vigor was the common response to natural stress and the difference in adaptability in hybrid and parental plants, among which the difference of photosynthesis was the most important (Ye and Wang, 2002). The photosynthetic capacity *Sorghum bicolor* × *S. sudanense*, which was derived from *S. sudanense* (Piper) Stapf and *S. bicolor* (L.) Moench, had a higher photosynthetic capacity than *S. sudanense* as well as better adaptability under drought stress (Zhang et al., 2012). The hybrid F_1_ generation of *Ipomopsis aggregate* × *Ipomopsis tenuttuba* showed higher hybrid vigor in the photosynthetic capacity at different phases than parental plants (Campell et al., 2005). The adaptability of the hybrid variety derived from *Iris fulva* and *Iris hexagona* to stress was obviously better than the parental plants (Burke et al., 1998). The hybrid offspring of *Helianthus annuus* and *H. petiolaris* also had a higher adaptability than the parental plants (Whitney et al., 2010).

Although *Physocarpus opulifolius* “Diabolo” has morphological and reproductive advantages, its stress resistance is relatively weak. In the cold areas of Northern China, its spring green up is relatively slow and its drought resistance is relatively lower than *P. amurensis* (Xu et al., 2017). Hybrid plants a have stronger photosynthetic capacity, which is one of the main aspects of hybrid vigor (Küppers et al., 2017; Wang et al., 2018). During 2006–2009, Yu et al. (2010) from the Forest Botanical Garden of Heilongjiang Province successfully obtained 202 plants of F_1_ seedlings from the hybrid of local *P. amurensis* (♀) × *P. opulifolius* “Diabolo” (♂), among which 88 plants had seedling leaf color identical or similar to the F_1_ hybrid *P. amurensis* of parental plants. The 88 F_1_ *P. amurensis* hybrid individuals not only had the ornamental quality desired of *P. opulifolius* “Diabolo” but also grew vigorously with a uniform canopy, presented stronger cold tolerance, and the development of hibernacles was drastically earlier than that of local *P. amurensis*. Our previous studies have found that although *P. opulifolius* “Diabolo” had good ornamental qualities, its salt tolerance was significantly lower than that of local *P. amurensis*. Compared with *P. amurensis* with stronger salt tolerance, it remains a question whether the obtained *P. opulifolius* hybrid with purple leaves had an advantage regarding salt tolerance. Therefore, by selecting cutting seedlings of the F_1_ generation of hybrid *P. amurensis* with better growth and purple leaves as experiment material, and the native *P. amurensis* (♀) and the imported *P. opulifolius* “Diabolo” (♂) from North America as controls.

Photosynthesis is the foundation of guaranteeing normal plant growth and development under environmental stress. Salt stress will inhibit plant growth through influencing the photosynthetic capacity of plants (Satoh et al., 1983; Ma et al., 1997). For example, the photosynthetic capacity of glycophyte plants, such as barley and wheat, was obviously inhibited by salt stress (Yang et al., 2008b, 2009). However, the photosynthetic capacity of the halophyte *Chloris virgata* was not impeded by higher salt-alkali stress (Yang et al., 2008a). Salt stress will inhibit the activity of the PSII reaction center in plant leaves. For example, the declining activity of OEC on the PSII electron donor side and degradation of proteins on the PSII electron receptor side will influence the electron transfer on the PSII acceptor side (Sharkey and Badger, 1982; Zhang et al., 2016a). The decrease of the electron transport rate will result in the accumulation of surplus electrons in the electron transport chain; thereafter, electron leak will attack free oxygen molecules in the cells and lead to burst out of reactive oxygen species (ROS) and also accelerate the degree of damage of the PSII reaction center (Percey et al., 2016; Hou et al., 2017), or even lead to the peroxidation or dissociation of thylakoid membranes (Mitsuya et al., 2000). Though numerous studies have been conducted on the photosynthesis under salt stress conditions, the influence of salt stress on the photosynthetic function of the hybrid *P. amurensis*, the native *P. amurensis* (♀), and the imported *P. opulifolius* “Diabolo” (♂) has not been studied. Here, we investigate whether the species that has stronger salt resistance will maintain higher photosynthetic ability, and if the damage or inhibition caused by salt stress on the taxa vary, such as if the stomatal and non-stomatal limitation of photosynthesis and the damage to the donor side and acceptor side of PSII under salt stress are different in the different salt resistant species. Clarifying these questions will increase our understanding of the mechanism of salt resistance in *P. amurensis* as well as the effects of salt stress on the photosynthetic function of these three taxa. Under salt stress, maintaining higher photosynthetic capacity is a manifestation of salt tolerance in plants. The relationship between two parental seedlings and a hybrid that whether their salt tolerance is consistent with the photosynthetic capacity. These results can provide a quick and convenient index for the screening of salt resistant plants in future studies.

## Materials and Methods

### Experiment Materials and Treatments

The experiment was conducted in the plant-physiology laboratory at the Northeast Forestry University from July to September 2016, and the experiment materials were 3-year-old cutting seedlings of triennial *P. amurensis, P. opulifolius* “Diabolo,” and the F_1_ hybrid *P. amurensis* (♀) × *P. opulifolius* “Diabolo” (♂) with purple leaves, which were provided by the Heilongjiang Forest Botanical Garden. The two parents and the F_1_ hybrid had three to five branches and were about 0.3–0.5 m. The seedlings were planted in plastic flower pots with an opening diameter of 28 cm, bottom diameter of 15 cm, and height of 20 cm. Each pot was planted with one plant and the cultivating matrix was turfy soil. On July 20th, 2016, the three sample types were irrigated with solutions containing four different concentrations of NaCl, 0 (CK), 50, 100, and 200 mmol⋅L^-1^ to conduct the salt stress tests (the selection of NaCl concentration according to Wang and Zhao, 2004). Each pot was irrigated with 1 L of solution and one tray was connected with each pot so that the percolated NaCl solution can be returned to the pot in time. One week after the salt treatment, the photosynthetic gas exchange parameters and chlorophyll fluorescence parameters were measured after varying salt damage in leaves was apparent.

#### The Measurement of Photosynthetic Gas Exchange Parameters

At 10:00 a.m. 1 week after the salt treatment, the Li-6400 photosynthesis measurement system (Licor, United States) was utilized to fix the light intensity at 1000 μmol⋅m^-2^⋅s^-1^ and CO_2_ concentration at 400 μL⋅L^-1^ for the simulation of these two indexes. In order to avoid a variation in the determination of photosynthetic indexes caused by different photosynthetic intensities and CO_2_ concentrations, uniform environmental parameters were selected. For each treatment, the third or fourth last fully expanded leaf on the current-year new shoot was selected to measure the photosynthetic gas exchange parameters such as net photosynthetic rate (*P*_n_), stomatal conductance (*G*_s_), transpiration rate (*T*_r_), and cellular CO_2_ concentration (*C*_i_).

#### The Measurement of Chlorophyll Fluorescence Parameters

The third or fourth last fully expanded leaf of *P. amurensis* Maxim seedlings from different treatments were processed with 0.5 h of dark adaptation using the dark adaptation clamps. Following the methods described in Hu et al. (2007), a portable pulse modulated fluorometer FMS-2 (Hansatech Co., United Kingdom) was utilized to measure the PSII maximum photochemical efficiency (*F*_v_/*F*_m_), actual photochemical efficiency (*Φ*_PSII_), photochemical quenching coefficient (*q*_P_), and non-photochemical quenching (NPQ). *F*_v_/*F*_m_ = (*F*_m_ -*F*_o_)/*F*_m_, *Φ*_PSII_ = (*F*_m_′ - *F*_s_)/*F*_m_′, *q*p = *(F*_m_′ -*F*_s_)/(*F*_m_′ -*F*_o_′), NPQ = *F*_m_*/F*_m_′ - 1. *F*_v_/*F*_m,_ *Φ*_PSII_, *q*_P_ and NPQ were calculated according to Genty et al. (1989). The measurement for each treatment was repeated three times.

#### The Measurement of Fast Chlorophyll Fluorescence Induction Kinetics Curve

The Handy-PEA continuous stimulus fluorometer (Handy, United Kingdom) was utilized to measure the fast chlorophyll fluorescence induction kinetics curve (OJIP fluorescence induction curve) of the 3rd or 4th last fully expanded leaf of different plants. Before measurement, the leaves were processed with dark adaptation for 30 min. The four characteristic points, O, J, I, and P, were the points corresponding to moment 0, 20, 30, and 1,000 ms on the OJIP curve, and their corresponding relative fluorescence intensities were denoted with *F*_o_, *F*_J_, *F*_i_, and *F*_m_, respectively. The 0.15 and 0.3 ms moments on the OJIP curve were defined as L and K, and their corresponding relative fluorescence intensity were denoted with *F*_L_ and *F*_k_, respectively. The standardization of the OJIP curves of different treatments should be processed with O–P, O–J, and O–K; the relative fluorescence intensity at point O was defined as 0, and point P, J, and K were defined as 1 for standardization. The standardization equation was *V*_O-P_ = (*F*_t_ -*F*_o_)/(*F*_P_ -*F*_o_), *V*_o-J_ = (*F*_t_ -*F*_o_)/(*F*_J_ -*F*_o_), and *V*_o-k_ = (*F*_t_ -*F*_o_)/(*F*_k_ -*F*_o_), respectively. In the equations above, *F*_t_ was the relative fluorescence intensity at different time points, and the three characteristic points L, K, and J of the standardized curves were denoted with *V*_L_, *V*_k_, and *V*_J_, i.e., *V*_L_ = (*F*_L_ -*F*_o_)/(*F*_k_ -*F*_o_), *V*_k_ = (*F*_k_ - *F*_o_)/(*F*_J_ -*F*_o_), and *V*_J_ = (*F*_J_ -*F*_o_)/(*F*_P_ -*F*_o_), respectively. The difference was calculated between the *V*_o-P_, *V*_o-J_, and *V*_o-k_ curves of plant leaves under different salt concentrations, and the CK curve, which was indicated as Δ*V*_o-P_, Δ*V*_o-J_, and Δ*V*_o-k_, respectively (Strasser et al., 1995; Zhang et al., 2016b).

#### The Measurement of Leaf Water Potential, and Leaf Na and Cl Concentrations

The leaves were rinsed with tap water first and then deionized water. After blotting water with filter paper, the leaves were placed at 105°C for 2 h for fixing, and at 60°C for drying to constant weight. The dried samples were comminuted with a grinder and passed through 0.5 mm-sieves. Samples (0.10 g) were digested in concentrated H_2_SO_4_-H_2_O_2_. Na^+^ content was measured using a flame photometer (FP6410). Cl^-1^ content was determined by titration with AgNO_3_. Leaf water potential was determined using the PSYPRY water potential meter (WESCOR, United States).

### Data Processing Method

Each experiment was repeated three times. Excel (2003) and SPSS (22.0) software were employed to conduct statistical analyses, and two-way analysis of variance (two-way ANOVA) and Least significant difference (LSD) tests were employed to compare the difference among different data groups. Differences were considered significant if *P* ≤ 0.05.

## Results and Analyses

### The Effect of Salt Stress on the Photosynthetic Gas Exchange Parameters of Leaves of Three *Physocarpus amurensis*

**FIGURE 1:**
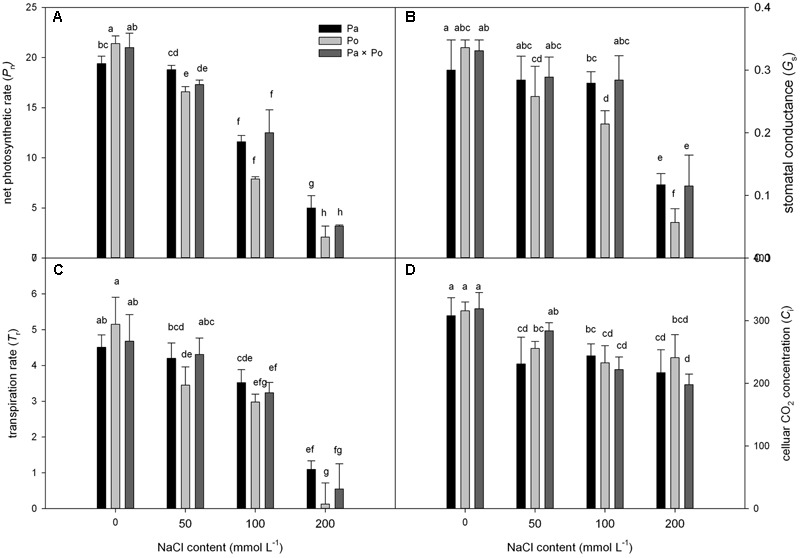
The effect of salt stress on the photosynthetic gas exchange parameters of leaves of three *Physocarpus amurensis*. Net photosynthetic rate **(A)**, stomatal conductance **(B)**, transpiration rate **(C)**, and celluar CO_2_ concentration **(D)**. Note: Pa, *P. amurensis* Maxim (♀); Po, *P. opulifolius* “Diabolo” (♂); Pa × Po, *P. amurensis* Maxim (♀) × *P. opulifolius* “Diabolo” (♂). Data in the figure are mean ± SE; values followed by different lowercase letters indicate a significant difference (*p* < 0.05).

Under non-salt stress, the *P*_n_, *G*_s_, and *T*_r_ of *P. opulifolius* “Diabolo” leaves were all slightly higher than those of *P. amurensis* and hybrid *P. amurensis* (**Figures 1A–D**). According to the two-way ANOVA analysis we found that *P*_n_, *G*_s_, *T*_r_, and *C*_i_ were significantly affected by the genotype and salt treatment, genotype × salt had significant interaction effects on *P*_n_ and *G*_s_ parameters of leaves from the three *P. amurensis* taxa, but no significant interaction with *T*_r_ and *C*_i_ (**Table 1**). However, with the increasing salt concentration, the decreasing rate of the *P*_n_, *G*_s_, and *T*_r_ of *P. opulifolius* leaves was obviously greater than that of *P. amurensis* and hybrid *P. amurensis*. Moreover, *P*_n_, *G*_s_, and *T*_r_ of *P. amurensis* and hybrid *P. amurensis* showed no significant difference under different salt concentrations. Overall, with the increase of salt concentration, the *C*_i_ of the leaves from the three *Physocarpus* taxa presented a declining trend and the difference between different varieties was relatively small. Yet, the *C*_i_ of *P. opulifolius* leaves under salt stress of 200 mmol⋅L^-1^ increased slightly compared with that under stress of 200 mmol⋅L^-1^.

**Table 1:** Two-way ANOVA examining the effects of genotype, saline treatment and their interaction (genotype × saline treatment) on the photosynthetic gas exchange parameters of leaves from the three *Physocarpus amurensis* sample types.

	Genotype		Saline treatment		Genotype × saline treatment	
	*F*	*P*	*F*	*P*	*F*	*P*
*P*_n_	619.83	<0.001	62.654	<0.001	19.476	<0.001
*G*_s_	82.84	<0.001	12.04	<0.001	9.54	<0.001
*T*_r_	41.88	<0.001	4.72	<0.01	1.90	0.12
*C*_i_	23.70	<0.001	7.33	<0.01	1.85	0.13

### The Effect of Salt Stress on the Chlorophyll Fluorescence Parameter of Leaves of Three *Physocarpus amurensis*

**FIGURE 2:**
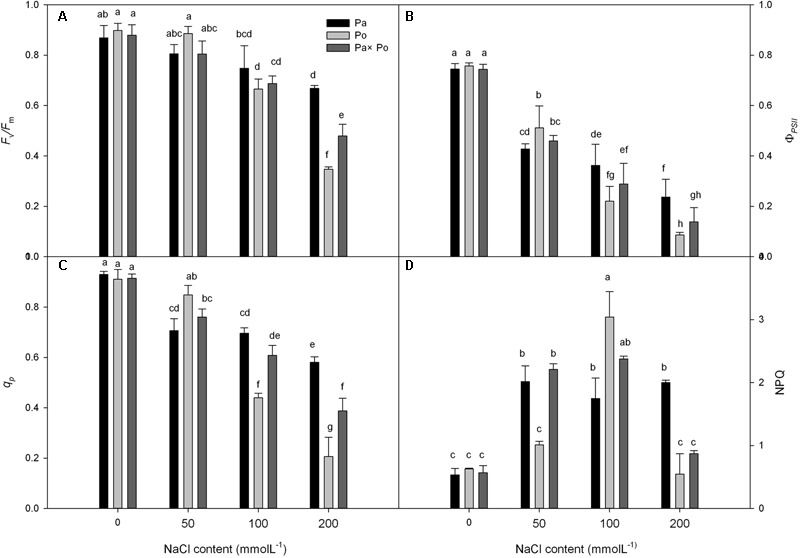
The effect of salt stress on the chlorophyll fluorescence parameters of leaves of three *P. amurensis*. The PSII maximum photochemical efficiency **(A)**, actual photochemical efficiency **(B)**, photochemical quenching coefficient **(C)**, and non-photochemical quenching **(D)**. Note: Pa, *P. amurensis* Maxim (♀); Po, *P. opulifolius* “Diabolo” (♂); Pa × Po, *P. amurensis* Maxim (♀) × *P. opulifolius* “Diabolo” (♂). Data in the figure are mean ± SE; values followed by different lowercase letters indicate a significant difference (*p* < 0.05).

Under non-salt stress conditions, the chlorophyll fluorescence parameters of leaves of the three *P. amurensis*t had no significant difference (**Figures 2A–D**). With the increase of salt concentration, *F*_v_/*F*_m_, *Φ*_PSII_, and *q*_P_ of leaves from the three *P. amurensis* taxa presented obvious declining trends, while the NPQ presented an overall trend of increasing first and then decreasing. Under salt concentrations of 50 mmol⋅L^-1^, the *F*_v_/*F*_m_, *Φ*_PSII_, and *q*_P_ of *P. opulifolius* “Diabolo” were slightly higher than those of *P. amurensis* and hybrid *P. amurensis*. The decreasing rates of *F*_v_/*F*_m_, *Φ*_PSII_, and *q*_P_ of *P. opulifolius* “Diabolo” under salt stress treatments of 100 and 200 mmol⋅L^-1^ were obviously greater than those of the two other taxa. In addition, the NPQ of the leaves of the three *P. amurensis*t under different concentrations of salt stress were all evidently higher than those not treated with salt stress. The NPQ of *P. opulifolius* “Diabolo” and hybrid *P. amurensis* leaves reached the highest under the concentration of 100 mmol⋅L^-1^, which significantly declined when the salt concentration rose to 200 mmol⋅L^-1^. However, the NPQ of *P. amurensis* did not show a decreasing trend, and the NPQ of *P. opulifolius* “Diabolo” under 200 mmol⋅L^-1^ decreased by 82.05% compared with that under 100 mmol⋅L^-1^, while the decrease of hybrid *P. amurensis* was smaller (63.33%) than that of *P. opulifolius* “Diabolo” (**Table 2**).

**Table 2:** Two-way ANOVA examining the effects of genotype, saline treatment and their interaction (genotype × saline treatment) on the chlorophyll fluorescence parameters of leaves from the three *P. amurensis* sample types.

	Genotype		Saline treatment		Genotype × saline treatment	
	*F*	*P*	*F*	*P*	*F*	*P*
*F*_v_/*F*_m_	54.11	<0.001	11.20	<0.001	3.61	0.01
Φ_PS_	292.50	<0.001	48.18	<0.001	7.57	<0.001
*q*_P_	125.79	<0.001	36.61	<0.001	9.19	<0.001
NPQ	18.70	<0.001	9.36	<0.001	11.89	<0.001

### The Effect of Salt Stress on the OJIP Curves of Leaves of the Three *Physocarpus amurensis*

**FIGURE 3:**
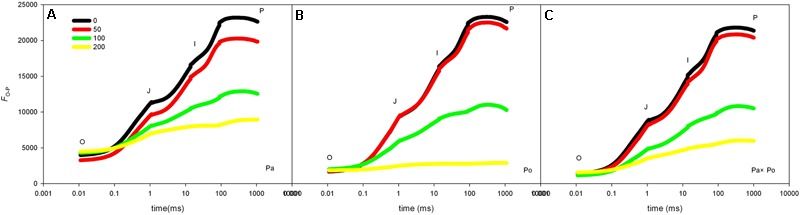
The effect of salt stress on the OJIP curves of leaves of the three *P. amurensis*. The effect of salt stress on the OJIP curves of *P. amurensis* **(A)**, the effect of salt stress on the OJIP curves of *P. opulifolius* “Diabolo” **(B)**, and the effect of salt stress on the OJIP curves of hybrid *P. amurensis* **(C)**. Note: Pa, *P. amurensis* Maxim (♀); Po, *P. opulifolius* “Diabolo” (♂); Pa × Po, *P. amurensis* Maxim (♀) × *P. opulifolius* “Diabolo” (♂). Data in the figure are mean ± SE; values followed by different lowercase letters indicate a significant difference (*p* < 0.05).

Compared with the OJIP curve of CK, the OJIP curve patterns of leaves of the three *P. amurensis* under different concentrations of salt stress had evident changes, which mainly presented as the following: the relative fluorescence intensity at point J, I, and P declined with the rising salt concentration, among which the extent of the decline at point P was the greatest; however, the relative fluorescence intensity at point O of the leaves from the three *P. amurensis*t under different concentrations did not show significant changes. The extent of the decrease of the relative fluorescence intensity at point J, I, and P of *P. amurensis* treated with different salt concentrations was obviously smaller than those of *P. opulifolius* “Diabolo” and hybrid *P. amurensis*, and the decrease observed in *P. opulifolius* “Diabolo” was the greatest (**Figures 3A–C**).

**FIGURE 4:**
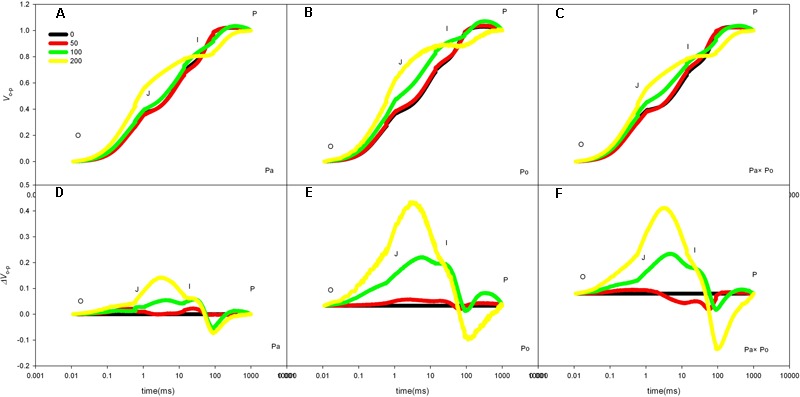
The effect of salt stress on the standardized OJIP curves of leaves of three *P. amurensis*. The effect of salt stress on the standardized OJIP curves of *P. amurensis* **(A,D)**. The effect of salt stress on the standardized OJIP curves of *P. opulifolius* “Diabolo” **(B,E)**. The effect of salt stress on the standardized OJIP curves of hybrid *P. amurensis* **(C,F)**. Note: Pa, *P. amurensis* Maxim (♀); Po, *P. opulifolius* “Diabolo” (♂); Pa × Po, *P. amurensis* Maxim (♀) × *P. opulifolius* “Diabolo” (♂). Data in the figure are mean ± SE; values followed by different lowercase letters indicate a significant difference (*p* < 0.05).

**FIGURE 5:**
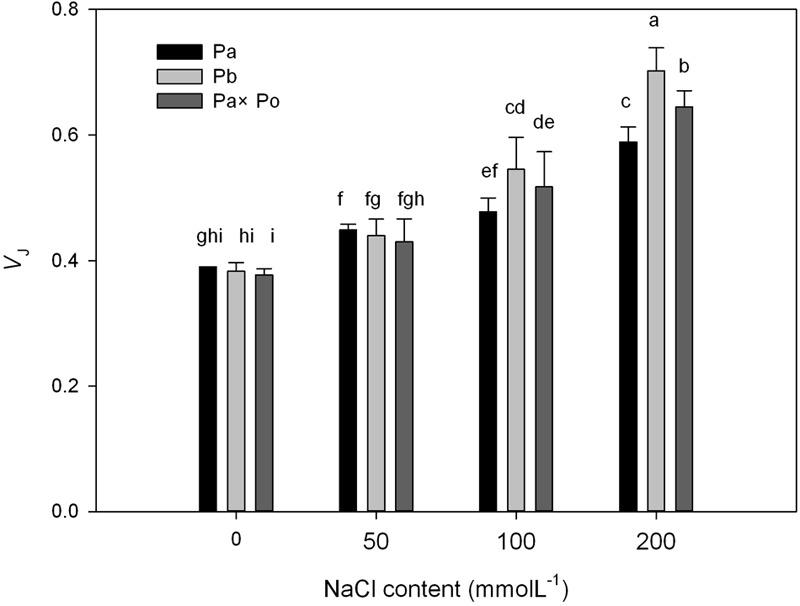
The effect of salt stress on the standardized OJIP curves and *V*_J_ of leaves of three *P. amurensis*. Pa, *P. amurensis* Maxim (♀); Po, *P. opulifolius* “Diabolo” (♂); Pa × Po, *P. amurensis* Maxim (♀) × *P. opulifolius* “Diabolo” (♂). Data in the figure are mean ± SE; values followed by different lowercase letters indicate a significant difference (*p* < 0.05).

After standardizing the OJIP curves of leaves of the three *P. amurensis* under different treatments (**Figures 4A–C**), we found that compared with the CK treatment, the standardized OJIP curves of *P. amurensis* under the salt concentrations of 50 and 100 mmol⋅L^-1^ did not change significantly. However, when salt increased to 200 mmol⋅L^-1^, the relative variable fluorescence at point J increased, while that of *P. opulifolius* “Diabolo” and hybrid *P. amurensis* increased when the salt concentration reached 100 mmol⋅L^-1^, and the extent of the increase of hybrid *P. amurensis* was slightly lower than that of *P. opulifolius* “Diabolo.” Through calculating the difference between standardized OJIP curves under different concentrations and the CK curve (**Figures 4D–F**), it could be seen that the relative variable fluorescence at various points of the three *P. amurensis* taxa under salt stress all were the highest at point J, and the extent of the increase was as follows: *P. opulifolius* “Diabolo” > hybrid *P. amurensis* > *P. amurensis*. The quantitative analysis of V_J_ under different salt concentrations (**Figure 5**) showed that under salt concentrations of 0 and 50 mmol⋅L^-1^, the V_J_ of leaves of the three *P. amurensis* taxa had no significant difference; however, under 100 and 200 mmol⋅L^-1^, the V_J_ of *P. amurensis* was 12.41% (*P* < 0.05) and 6.18% (*P* < 0.05) lower than that of *P. opulifolius* “Diabolo” and hybrid *P. amurensis*, respectively, and these differences are significant. Although the V_J_ of hybrid *P. amurensis* was also higher than that of *P. amurensis*, the difference was not significant (**Table 3**).

**Table 3:** Two-way ANOVA examining the effects of genotype, saline treatment and their interaction (genotype × saline treatment) on the *V*_J_ of leaves from the three *P. amurensis* sample types.

	*F*	*P*
Genotype	23.58	<0.001
Saline treatment	3.05	<0.001
Genotype × saline treatment	6.41	<0.001

### The Effect of Salt Stress on the Standardized O–J Curves and V_k_ of Leaves of the Three *Physocarpus amurensis*

**FIGURE 6:**
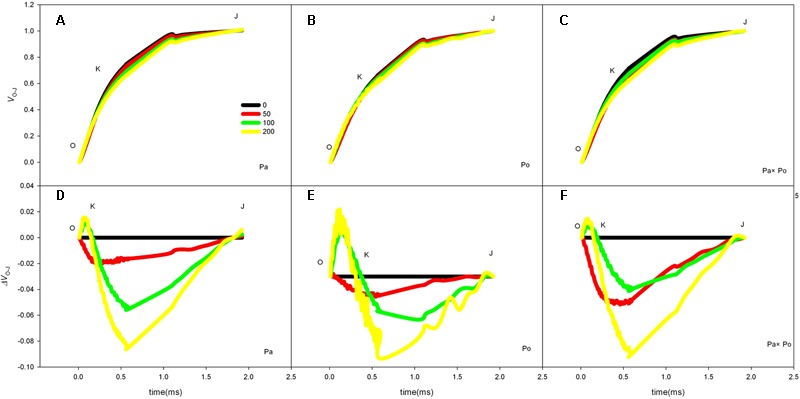
The effect of salt stress on the standardized O–J curves and *V*_k_ of leaves from the three *P. amurensis* sample types. Pa, *P. amurensis* Maxim (♀); Po, *P. opulifolius* “Diabolo” (♂); Pa × Po, *P. amurensis* Maxim (♀) × *P. opulifolius* “Diabolo” (♂). Data in the figure are mean ± SE; values followed by different lowercase letters indicate a significant difference (*p* < 0.05).

**FIGURE 7:**
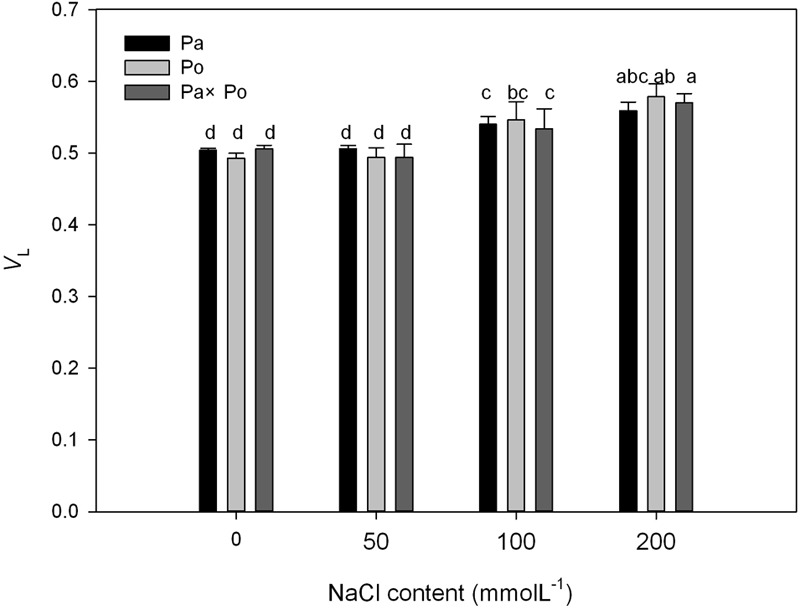
The effect of salt stress on the standardized O–J curves and *V*_k_ of leaves from the three *P. amurensis* sample types. Pa, *P. amurensis* Maxim (♀); Po, *P. opulifolius* “Diabolo” (♂); Pa × Po, *P. amurensis* Maxim (♀) × *P. opulifolius* “Diabolo” (♂). Data in the figure are mean ± SE; values followed by different lowercase letters indicate a significant difference (*p* < 0.05).

By defining the relative fluorescence intensity of point O as 0 and that of point J as 1, we investigated the O–J standardization of the curves of the three *P. amurensis* (**Figures 6A–C**), and then subtracted the treatment results with the control (**Figures 6D–F**). We found that under salt stress, the extent of change of standardized O–J curves of the three *P. amurensis* sample types was relatively small compared with that of the CK. In comparison with the CK, at the point of about 0.5 ms of the O–J curve, the relative variable fluorescence (*V*_k_) obviously decreased while that at the characteristic point of 0.3 ms. The extent of change was small, and only the *V*_k_ of *P. opulifolius* “Diabolo” under salt concentrations of 100 and 200 mmol⋅L^-1^ increased to some degree (**Figure 7**). Quantitative analysis of the change in *V*_k_ of the three *P. amurensis* varieties under salt stress showed that, under non-salt stress, the *V*_k_ of *P. opulifolius* “Diabolo” leaves was significantly lower than that of *P. amurensis* and hybrid *P. amurensis*. However, with the increasing salt concentrations, the *V*_k_ of *P. amurensis* and hybrid *P. amurensis* obviously declined while that of *P. opulifolius* “Diabolo” increased a little, and the difference under different salt concentrations was not significant (**Table 4**).

**Table 4:** Two-way ANOVA examining the effects of genotype, saline treatment and their interaction (genotype × saline treatment) on the *V*_k_ of leaves from the three *P. amurensis* sample types.

	*F*	*P*
Genotype	18.86	<0.001
Saline treatment	4.83	<0.01
Genotype × saline treatment	13.79	<0.001

### The Effect of Salt Stress on the Standardized O–K Curves and *V*_L_ of Leaves From Three *Physocarpus amurensis* Sample Types

**FIGURE 8:**
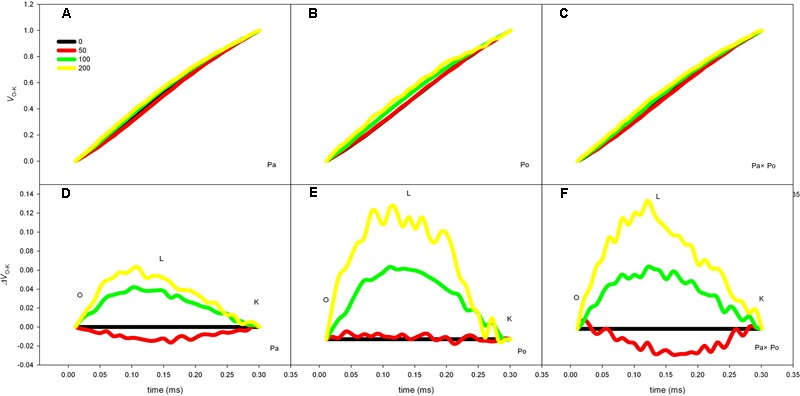
The effect of salt stress on the standardized O–K curves of leaves from three *P. amurensis* sample groups. The effect of salt stress on the standardized O–K curves of *P. amurensis* **(A,D)**. The effect of salt stress on the standardized O–K curves of *P. opulifolius* “Diabolo” **(B,E)**. The effect of salt stress on the standardized O–K curves of hybrid *P. amurensis* **(C,F)**. Note: Pa, *P. amurensis* Maxim (♀); Po, *P. opulifolius* “Diabolo” (♂); Pa × Po, *P. amurensis* Maxim (♀) × *P. opulifolius* “Diabolo” (♂). Data in the figure are mean ± SE; values followed by different lowercase letters indicate a significant difference (*p* < 0.05).

**FIGURE 9:**
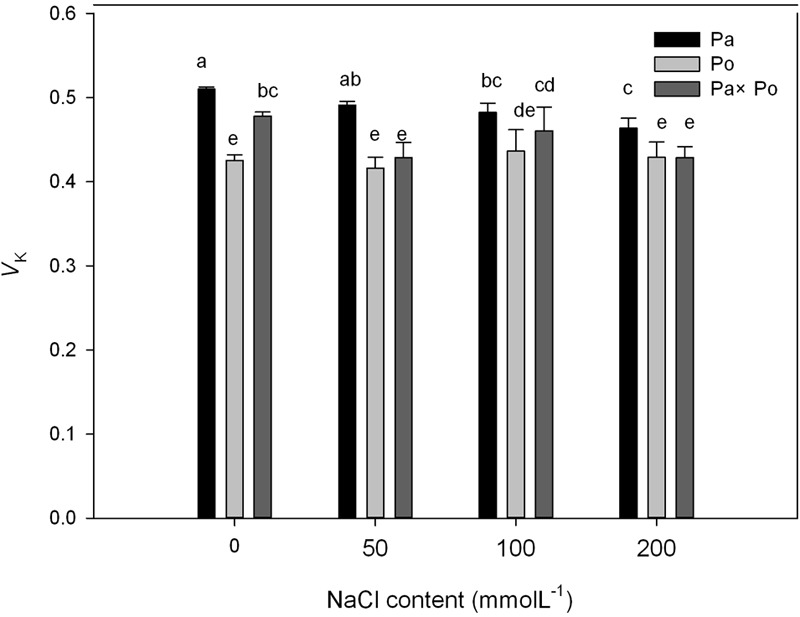
The effect of salt stress on the standardized O–K curves and *V*_L_ of leaves from three *P. amurensis* sample groups. Pa, *P. amurensis* Maxim (♀); Po, *P. opulifolius* “Diabolo” (♂); Pa × Po, *P. amurensis* Maxim (♀) × *P. opulifolius* “Diabolo” (♂). Data in the figure are mean ± SE; values followed by different lowercase letters indicate a significant difference (*p* < 0.05).

The *P. amurensis* samples under salt stress had relative variable fluorescence (*V*_L_) at 0.15 ms, i.e., point L of the standardized O–K curves presented evident differences (**Figures 8A–C**). By calculating the difference between the standardized O–K curves and CK curves of the three *P. amurensis* under salt stress (**Figures 8D–F**), the *V*_L_ of *P. amurensis* and hybrid *P. amurensis* under a salt concentration of 50 mmol⋅L^-1^ decreased slightly while that of *P. opulifolius* “Diabolo” did not change. However, under salt concentrations of 100 and 200 mmol⋅L^-1^, *V*_L_ of leaves from three *P. amurensis* sample types all increased, and the increase in *P. opulifolius* “Diabolo” was the greatest. Under salt concentrations of 100 and 200 mmol⋅L^-1^, the quantitative analysis of the change of *V*_L_ indicated that the *V*_L_ of *P. opulifolius* “Diabolo” leaves was slightly higher than that of *P. amurensis* and hybrid *P. amurensis*, and their difference was not significant (**Figure 9** and **Table 5**).

**Table 5:** Two-way ANOVA examining the effects of genotype, saline treatment and their interaction (genotype × saline treatment) on the *V*_L_ of leaves from the three *P. amurensis* sample types.

	*F*	*P*
Genotype	45.96	<0.001
Saline treatment	10.36	<0.001
Genotype × saline treatment	2.71	0.03

### The Effect of Salt Stress on the Leaf Water Potential, Na^+^ and Cl^-^ Concentrations

**FIGURE 10:**
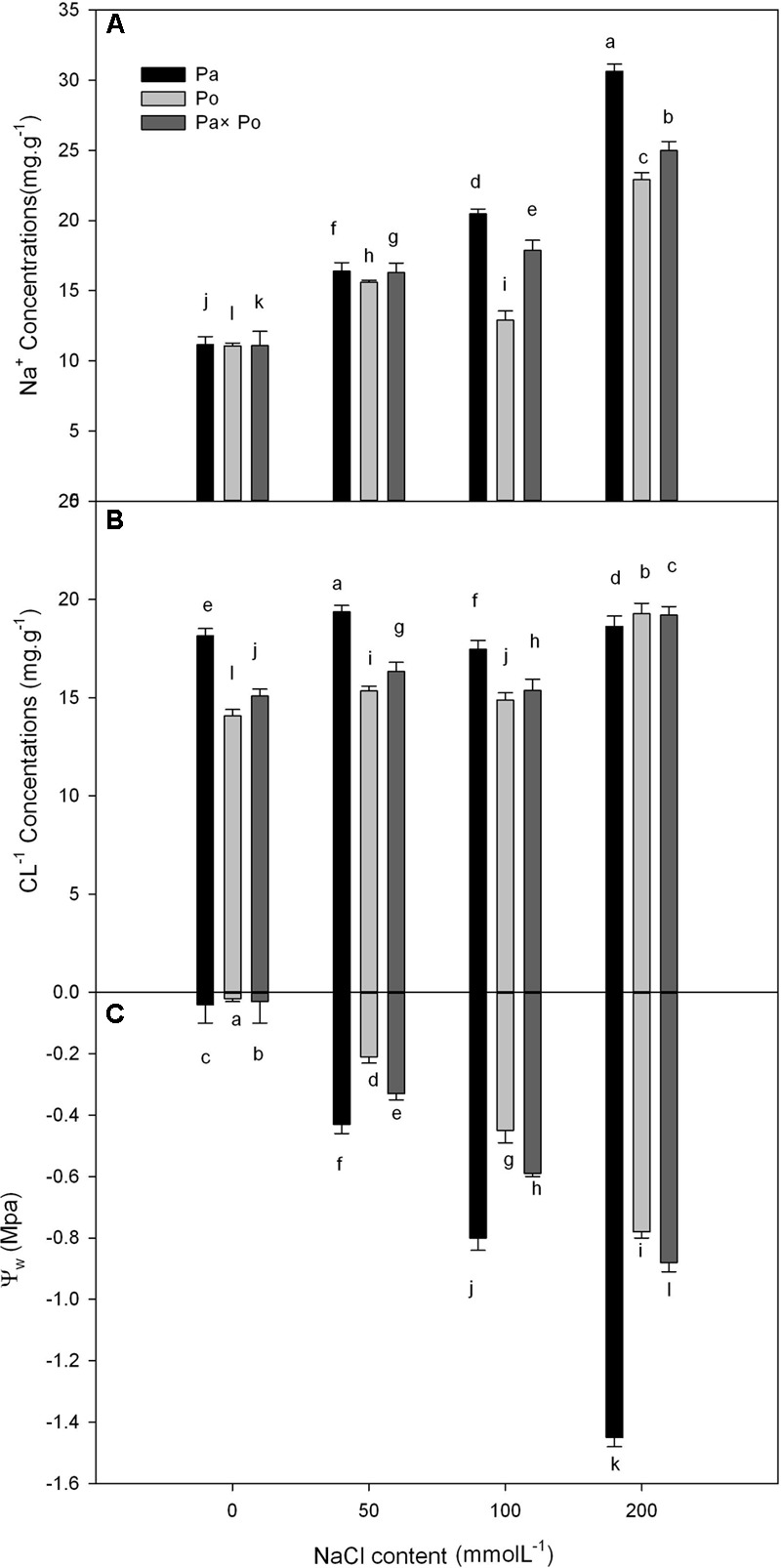
The effect of salt stress on the leaf water potential, and leaf Na^+^ and Cl^-^ concentrations of leaves from three *P. amurensis* sample groups. Na^+^ concentrations of leaves from three *P. amurensis* sample groups **(A)**. Cl^-^ concentrations of leaves from three *P. amurensis* sample groups **(B)**. Water potential of leaves from three *P. amurensis* sample groups **(C)**. Note: Pa, *P. amurensis* Maxim (♀); Po, *P. opulifolius* “Diabolo” (♁); Pa × Po, *P. amurensis* Maxim (♀) × *P. opulifolius* “Diabolo” (♁). Data in the figure are mean ± SE; values followed by different lowercase letters indicate a significant difference (*p* < 0.05).

With increasing NaCl concentration, Na^+^ concentration in the three experimental samples increased significantly, which was the most obvious in *P. amurensis* (increased by about 63.6%), followed by *P. opulifolius* (55.7%) and *P. amurensis hybrids* (51.7%). Leaf water potential decreased with the concentration of NaCl increased, with *P. amurensis* presenting the most apparent decrease (by 97.2%). (**Figure 10** and **Table 6**).

**Table 6:** Two-way ANOVA examining the effects of genotype, saline treatment and their interaction (genotype × saline treatment) on the leaf water potential, Na^+^ and Cl^-^ concentrations of leaves from the three *P. amurensis* sample types.

	Genotype		Saline treatment		Genotype × saline treatment	
	*F*	*P*	*F*	*P*	*F*	*P*
Leaf water potential	627.53	<0.001	60.63	<0.001	90.94	<0.001
Na^+^ concentration	19.683	<0.001	7.726	<0.001	18.84	<0.001
Cl^-^ concentration	243.56	<0.001	144.24	<0.01	317.33	<0.001

## Discussion

Photosynthesis is sensitive to stress (Chaves et al., 2009). With the increasing salt concentration, the stomatal conductance of leaves from three *P. amurensis* sample groups declined, which not only resulted in the decrease of the transpiration rate of leaves but also reduced the net photosynthetic rate of leaves; specifically, the photosynthetic carbon assimilation capacity was inhibited. Under different salt concentrations, the extent of the decrease of *P*_n_, *G*_s_, and *T*_r_ of *P. opulifolius* “Diabolo” and hybrid *P. amurensis* leaves were obviously greater than those of *P. amurensis*, which indicated that the salt tolerance of photosynthetic characteristics of *P. opulifolius* “Diabolo” was lower than that of *P. amurensis*. Although the photosynthetic gas exchange parameters of hybrid *P. amurensis* leaves were lower than those of *P. amurensis*, they were higher than those of *P. opulifolius* “Diabolo.” In addition to salt ions directly inducing stomatal closure, stomatal factors were also connected with physiological drought resulting from water potential decreasing in the matrix induced by salt stress. The non-stomatal factors might be the toxicity of salt ions reducing the CO_2_ assimilation capacity in mesophyll cells and resulting in the accumulation of cellular CO_2_ (Chen et al., 2009; Zhang et al., 2012). In this study, when the salt concentration reached 200 mmol⋅L^-1^, although the *G*_s_ of the leaves from three *P. amurensis* sample groups obviously decreased, so did the *P*_n_, but the *C*_i_ did not change, indicating that under high salt stress the decrease of photosynthetic capacity was the result of the co-limitation of both the stomatal factors and non-stomatal factors. Under mild salt stress, the decrease of photosynthesis is mainly restricted by stomata, while it is mostly affected by mesophyll factors under severe salt stress. The *G*_s_ of *P. opulifolius* “Diabolo” leaves was obviously lower than that of *P. amurensis* and hybrid *P. amurensis*, but the difference in *C*_i_ among the three sample types was not significantly different, indicating that the reason for inducing the decrease of photosynthetic capacity of *P. opulifolius* “Diabolo” was mainly due to non-stomatal factors. Furthermore, the CO_2_ utilization capacity of *P. opulifolius* “Diabolo” mesophyll cells was sensitive to salt stress, while that of hybrid *P. amurensis* was improved to some extent.

Under salt stress, among all the non-stomatal factors, except for the CO_2_ utilization capacity of mesophyll cells leading to the decrease of photosynthetic capacity, the decrease of PSII reaction center activity was also thought to be an important factors limiting photosynthetic capacity under salt stress (Lu and Zhang, 2000). In this study, with the increase of salt concentration, *F*_v_/*F*_m_, *Φ*_PSII_, and *q*_P_ of leaves from the three *P. amurensis* all decreased significantly, indicating that under salt stress the PSII photochemical activity of the three sample groups declined and photo-inhibition was evident under high salt stress conditions. However, the degree of photo-inhibition of *P. amurensis* and hybrid *P. amurensis* under salt stress was obviously lower than that of *P. opulifolius* “Diabolo,” and the extent of the change of chlorophyll fluorescence parameters of hybrid *P. amurensis* leaves was also lower than in *P. opulifolius* “Diabolo,” this indicated that hybridization with the highly salt tolerant *P. amurensis* can evidently improve the salt tolerance of the PSII reaction center of hybrid *P. amurensis* leaves. The formation of triplet state excited-state chlorophyll was inhibited by the xanthophyll cycle, and NPQ was positively correlated with heat dissipation depending on the xanthophyll cycle (Li et al., 2000). In this study, the NPQ of *P. amurensis* leaves under different salt concentrations were all higher than that of the CK treatment, i.e., *P. amurensis* can reduce the accumulation of excessive light energy within leaves under salt stress through increasing the xanthophyll cycle. However, the NPQ of *P. opulifolius* “Diabolo” and hybrid *P. amurensis* presented a trend of increasing first and then decreasing with the increasing salt concentration, indicating that under low salt concentrations *P. opulifolius* “Diabolo” and hybrid *P. amurensis* can release the surplus light energy in time through starting the heat dissipation mechanism depending on the xanthophyll cycle, but the protective effect decreased under high salt stress. The NPQ of hybrid *P. amurensis* leaves under different salt concentrations were all higher than those of *P. opulifolius* “Diabolo,” which indicated that the defensive mechanism of photo-damage of hybrid *P. amurensis* leaves under salt stress was increased compared with *P. opulifolius* “Diabolo.”

The fast chlorophyll fluorescence kinetics curve was utilized to analyze the injured PSII site of the three *P. amurensis* taxa. The increase of the relative variable fluorescence at point K of 0.3 ms (*V*_k_) has been shown to indicate that the oxygen-evolving complex (OEC) on the PSII electron donor side was injured (Zhang et al., 2012, 2016a,b, 2017). The function of the OEC is mainly involved in water splitting and oxygen release (Guskov et al., 2009), and when the expression quantity of the OEC decreases or its activity declined, the PSII activity of plant leaves decreases (Brandle et al., 1977). The OEC activity and expression quantity of proteins have been shown to be affected by salt stress (Abbasi and Komatsu, 2004; Park et al., 2004). In this study, the increase of VK under salt stress indicated that the OEC of plant leaves was damaged under the increasing salt concentrations. Research has shown that the expression of the core proteins from the OEC decrease under salt stress (Allakhverdiev et al., 2001; Pang et al., 2010). Our experimental results showed that with the increasing salt concentration, the *V*_k_ of *P. opulifolius* “Diabolo” leaves increased slightly, but the *V*_k_ of *P. amurensis* and hybrid *P. amurensis* leaves presented a decreasing trend (**Figure 5**), which indicated that the effect of salt stress on the OEC of leaves from the three *P. amurensis* sample types was relatively small and it even enhanced the OEC activity of *P. amurensis* and hybrid *P. amurensis*. Therefore, the enhancement of OEC activity of *P. amurensis* leaves resulting from the salt stress from NaCl was likely related with its Cl^-^ absorption. This result was consistent with the results presented by Pang (Pang et al., 2010) and that the OEC protein expression in *Arabidopsis, Thellungiella halophile*, and *H. tuberosus* L. was up-regulated under salt stress.

Under salt stress, the blocked sites of PSII electron transference usually occurred on the electron receptor side of the PSII reaction center, and the electron transfer from *Q*_A_ (the primary electron receptor of the electron transfer chain of Photosystem II) to *Q*_B_ (the secondary electron receptor of the electron transfer chain of Photosystem II) were the main inhibited sites under salt stress (Murata et al., 2007). The D_1_ protein was the PSII core protein combined with *Q*_B_, which had a fast turnover and its synthesis and degradation in the photosynthetic electron transfer chain remained in a dynamic balance; once the degradation rate was higher than the synthesis rate, net degradation of the D_1_ protein would occur and result in the decrease of *F*_v_/*F*_m_ (Takahashi and Murata, 2005). Under the adverse conditions, the Calvin cycle and removal mechanism of ROS were inhibited in plant cells; the ROS content in chloroplasts increased and then impeded the turnover of the D_1_ protein, leading to blockage of PSII electron transfer and the acceleration of photo-inhibition (Haldimann and Strasser, 1999; Cheng et al., 2016). The relative variable fluorescence at 2 ms of the OJIP curve, point J (*V*_J_) represented the degree of closure of the active reaction center and the increase of *V*_J_ indicated that the electron transfer from *Q*_A_ to *Q*_B_ in the photosynthetic electron transfer chain was inhibited and the accumulation of redox-state *Q*_A_ gradually increased (Venkatesh et al., 2012). In this study, with the increase of salt concentration, the *V*_J_ of leaves from the three *P. amurensis* sample groups all increased, specifically the salt stress evidently inhibited electron transfer from *Q*_A_ to *Q*_B_ on the PSII electron receptor side. With the increasing salt concentration, the extent of increase of *V*_J_ in *P. opulifolius* “Diabolo” was evidently greater than that of *P. amurensis*, suggesting that the electron transfer on the PSII electron receptor side of *P. opulifolius* “Diabolo” leaves was more sensitive to salt stress. However, the increase of *V*_J_ of hybrid *P. amurensis* was significantly lower than that of *P. opulifolius* “Diabolo,” which showed that the salt tolerance of the electron transfer on the PSII electron receptor side of hybrid *P. amurensis* was improved compared with *P. opulifolius* “Diabolo,” but whether it was related with the turnover of the D_1_ protein needs further verification.

When electron transfer on the PSII electron transfer chain was blocked, the accumulation of excessive electrons leads to electronic leak and the leaked electrons will attack free O_2_ within cells resulting in the formation of superoxide anions such as ROS (Chen et al., 2005). ROS are an important substance in plant cells and under normal physiological conditions, the ROS produced within cells can be removed rapidly by the scavenging system to prevent excessive oxidative damage to cells. However, under adverse conditions the intracellular ROS will usually accumulate and then damage cells with strong oxidation (Xu et al., 2000). The ROS mediated by photosynthesis will first attack thylakoid membranes to accelerate its degree of per-oxidation, and the peroxidation of thylakoid membranes will result in the decrease of PSII activity, blockage of electron transfer, and induction of the formation of more ROS, which forms a damaging cycle (Zhao et al., 2006; Nishiyama et al., 2011). The results of this study showed that salt stress could lead to the decrease of photochemical efficiency and NPQ, which causes the increase of excess excitation energy and ROS in the leaves, and the increased ROS would inhibit the synthesis of the D1 protein, aggravating photoinhibition (Mcconn and Browse, 1998; Yang et al., 2004). The increase of relative variable fluorescence of point L (*V*_L_) has been shown to indicate a change of thylakoid membrane fluidity, which is a major indicator that the structural and functional integrity of the thylakoid membrane has been destroyed (De Ronde et al., 2005; Tóth et al., 2005; Essemine et al., 2012). In this study, the *V*_L_ change of leaves from the three *P.s amurensis* sample groups under the salt stress of 50 mmol⋅L^-1^ was relatively small, and the *V*_L_ of *P. amurensis* and hybrid *P. amurensis* leaves decreased slightly compared with the control, which indicated that the effect of 50 mmol⋅L^-1^ of salt stress on the thylakoid membranes of the three sample groups was small. However, with the further increase of salt concentrations, the degree of damage to the thylakoid membranes of the three sample types increased, especially in the dissociation of the thylakoid membrane in *P. opulifolius* “Diabolo,” which was greater than that of *P. amurensis*, but the dissociation of the thylakoid membrane of hybrid *P. amurensis* was evidently smaller than that of *P. opulifolius* “Diabolo.”

We analyzed the leaf water potential, Na^+^ and Cl^-1^ concentrations of the three experimental samples, demonstrating that the water potential and Na^+^ concentration of the three samples changed significantly with the increase of salt stress. According to the definition of Levitt (1980), the salt concentration that is enough to reduce plant water potential (0.5–1 Mpa) will cause salt damage to plants. With the increasing concentration of NaCl, the leaf water potential of *P. amurensis* showed a significantly decreasing trend, suggesting that a continuous increase of salt stress caused salt damage to *P. amurensis* leaves. In our study *P. amurensis* showed higher salt tolerance in photosynthetic characteristics mainly involved in non-stomatal factors, although *P. amurensis* had higher leaf Na^+^ than the others (**Figure 10**). This suggests a greater capacity of Na^+^ compartmentation in the vacuoles in *P. amurensis*, which might be an underlying mechanism of salt tolerance in this species.

## Conclusion

The inhibition effect of salt stress on the photosynthesis of the three *P. amurensis* sample groups was the combined result of stomatal factors and non-stomatal factors. Among non-stomatal factors, the decrease of CO_2_ utilization capacity of mesophyll cells and limited activity of the PSII reaction center were both important reasons. Under high-concentrations of salt stress, *P. amurensis* can reduce the production of excessive light energy by increasing NPQ, while the NPQ of *P. opulifolius* “Diabolo” was evidently reduced under high salt stress. Salt stress obviously inhibited the electron transfer capacity from *Q*_A_ to *Q*_B_ on the PSII receptor side, and resulted in the dissociation of thylakoid membranes to some extent, but its harm to the OEC activity on the PSII donor side was relatively limited. The hybrid variety of *P. amurensis* (♀) × *P. opulifolius* “Diabolo” (♂) not only maintained the desired purple leaves of *P. opulifolius* “Diabolo,” but also inherited the stronger salt tolerance of the female parent, *P. amurensis*, and its salt tolerance in photosynthesis improved evidently compared with that of *P. opulifolius* “Diabolo.” In addition, further studies are required on the mechanism of photosynthetic function in hybrid *Physocarpus* leaves showing obvious heterosis, whether it is related to the stronger salt tolerance of roots, or the reduction of salt ion sensitivity by photosynthetic mechanism, or the enhanced scavenging capacity of active oxygen.

## Data Availability

The following information was supplied regarding data availability: The research in this article did not generate any raw data.

## Author Contributions

XN, ZHa, and ZHu conceived and designed the experiments. All authors performed the experiments and analyzed the data. XN and ZHu wrote the manuscript and prepared the figures and/or tables.

## Conflict of Interest Statement

The authors declare that the research was conducted in the absence of any commercial or financial relationships that could be construed as a potential conflict of interest.
